# Dual Oncological Challenges: Management of Simultaneous Lung Adenocarcinoma and Primary Cardiac Lymphoma

**DOI:** 10.7759/cureus.63396

**Published:** 2024-06-28

**Authors:** Andrew Strike, Michael Vidal, Palwasha Khan, Steven B Barker, Navneeth Bongu

**Affiliations:** 1 Graduate Medical Education, Northeast Georgia Medical Center Gainesville, Gainesville, USA; 2 Internal Medicine, Northeast Georgia Medical Center Gainesville, Gainesville, USA; 3 Pulmonary and Critical Care, Northeast Georgia Medical Center Gainesville, Gainesville, USA

**Keywords:** rituximab therapy, non-small cell lung cancer, multidisciplinary treatment, immunohistochemistry, biopsy, diagnostic imaging, diffuse large b-cell lymphoma, primary cardiac lymphoma, lung adenocarcinoma, concurrent primary cancers

## Abstract

Adenocarcinoma of the lung and primary cardiac lymphoma are both significant malignancies with serious health impacts. This case involves a 67-year-old woman who presented with progressive shortness of breath and fatigue. Initial computed tomography (CT) imaging identified possible cardiac and pulmonary masses, leading to her transfer to a specialized care center. Subsequent analysis confirmed adenocarcinoma of the lung, and further imaging and biopsy of the cardiac mass revealed diffuse large B-cell lymphoma. The patient received treatments targeted to each cancer, including chemotherapy and immunotherapy. This concurrence of malignancies highlights the importance of comprehensive diagnostic evaluations and personalized therapeutic strategies. Further research is needed to improve the management of patients with concurrent primary cancers.

## Introduction

Adenocarcinoma of the lung is the most common non-small cell lung cancer (NSCLC), accounting for nearly 40% of all lung cancer cases in males and more than 50% in females [1,2]. Pulmonary adenocarcinoma is often associated with tobacco use but can occur in non-smokers as well, with an increased prevalence in women non-smokers [3]. This malignancy can be asymptomatic in the early stages, often leading to delays in diagnosis and treatment. Common symptoms include chest pain, progressive dyspnea, and cough with hemoptysis. Weight loss and fatigue are also often present [4]. Diagnosis typically relies on initial imaging followed by tissue biopsy for histopathological and molecular testing [4].

Diffuse large B-cell lymphoma (DLBCL) is the most prevalent subtype of non-Hodgkin lymphoma, accounting for nearly 30% of lymphoma cases [5]. DLBCL is characterized by the aggressive growth of large lymphoid cells in both nodal and extranodal sites, with extranodal involvement present in less than half of cases at diagnosis [5]. The gastrointestinal tract is the most common site for extranodal involvement [6]. Primary cardiac lymphoma (PCL) is a very rare form of DLBCL, representing less than 2% of all cardiac tumors [7]. Symptoms are often very nonspecific, involving shortness of breath, chest pain, and other findings consistent with heart failure [7]. While imaging studies are often used to help identify potential PCL, definitive diagnosis requires biopsy and pathology.

## Case presentation

A 67-year-old woman with a history significant for tobacco use (with a 40-pack-year history) presented to a rural community hospital with progressive shortness of breath and fatigue. Her symptoms had been present for nearly six months. Additionally, she experienced a persistent cough that occasionally led to post-tussive hematemesis. Initial workup included a CT scan of the chest, which revealed a questionable cardiac mass and pulmonary infarct. Given the concern for these imaging findings, the patient was transferred to a tertiary care center for further evaluation and management.

Upon hospital transfer, the patient appeared fatigued but denied any acute distress. Her vital signs were significant for elevated blood pressure at 141/94 mmHg, tachycardia at 108 beats per minute, and elevated respiratory rate at 22 breaths per minute. Physical examination revealed decreased breath sounds in the right base. The patient was saturating appropriately on 2 liters of supplemental oxygen, but minimal movement led to respiratory distress.

**Figure 1:**
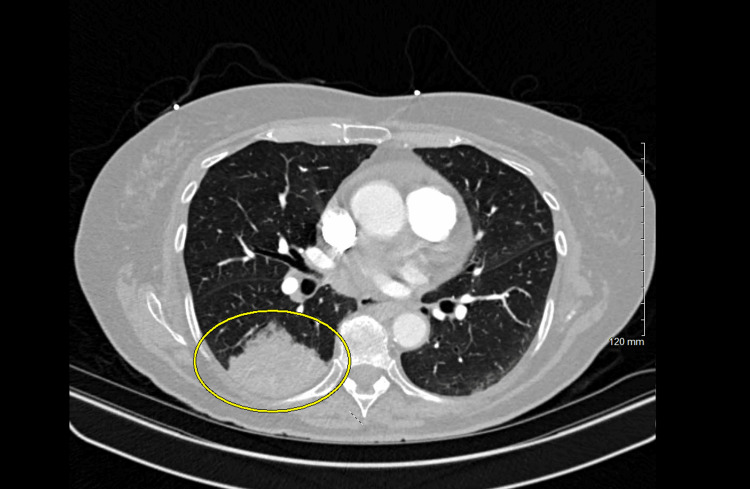
Wedge-shaped mass of the upper lobe of the right lower lung, later identified as adenocarcinoma

After admission, the admitting hospitalist team reviewed the outside CT scan. Imaging revealed a dense focus of peripheral wedge-shaped consolidation within the superior segment of the right lower lobe, measuring 45 x 60 mm in size. The findings raised suspicion for malignancy given the patient’s significant smoking history. CT-guided biopsy of the mass was completed. Figure 1 shows the CT scan of the lungs with the concerning findings).

After a successful biopsy, pathology revealed a mucin-producing malignant neoplasm with gland formation consistent with pulmonary adenocarcinoma. Neoplastic cells were positive for cytokeratin 7 and thyroid transcription factor-1 (TTF-1). A follow-up positron emission tomography (PET) scan showed high metabolic activity in the area of the lung mass, supporting the diagnosis. Of note, the PET scan did not show any signs of metastasis or other areas of uptake. Given the size of the mass and lack of mediastinal adenopathy or distant metastatic disease, the adenocarcinoma of the lung was staged at T3N0Mx.

**Figure 2:**
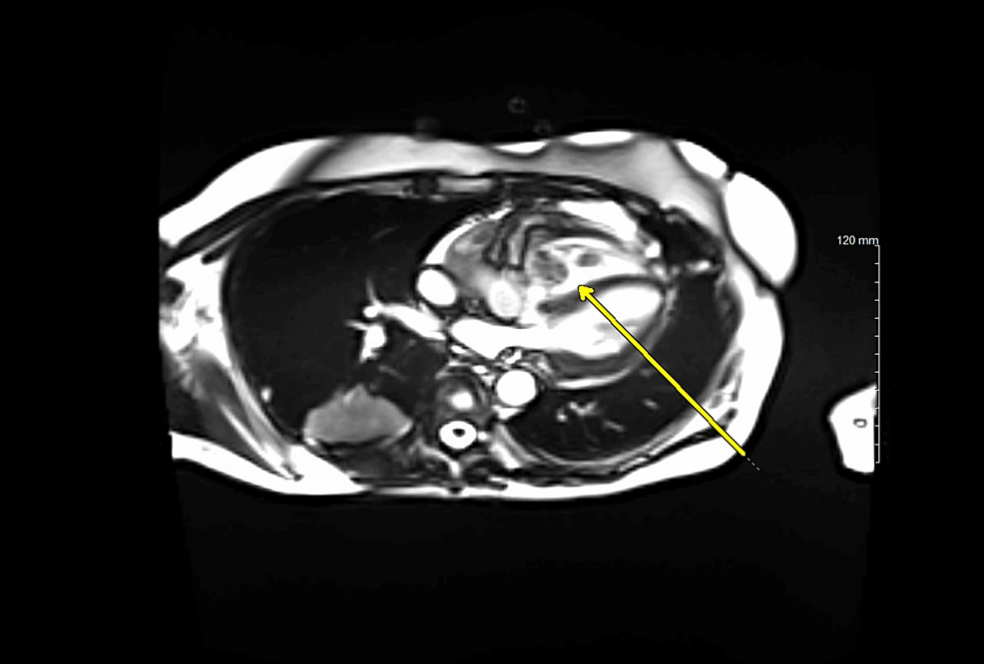
Multilobular right ventricular mass on cardiac MRI, later identified as diffuse large B-cell lymphoma

Further imaging was completed to explore the cardiac mass seen on initial imaging. The outside hospital CT showed a right ventricular mass measuring 5.5 x 4.6 cm, with concern for compression at the tricuspid valve without signs of tamponade. Cardiac magnetic resonance imaging (MRI) was completed, revealing irregular borders and heterogeneous enhancement of the mass, suggestive of malignancy. The mass was multilobulated with a highly mobile superior lobe and diffuse vascularization. Figure 2 shows the MRI findings.

After completing the imaging and lung biopsy, the patient was discharged with supplemental oxygen for home use. She was referred to outpatient oncology at another large academic center, where the patient was treated for pulmonary adenocarcinoma with intravenous (IV) cisplatin, pemetrexed, and nivolumab.

During outpatient follow-up with oncology, cardiothoracic surgery was consulted to complete a cardiac mass biopsy. The biopsy was sent for pathology, which showed diffuse infiltration of large lymphoid cells that tested positive for CD45, CD20, MUM1, Pax5, and BCL6. Surprisingly, a bone marrow biopsy was benign, with follow-up CT imaging showing no other signs of lymphoma involvement. Findings were consistent with isolated extranodal (IE) DLBCL of the right ventricle.

After completion of her extensive workup, the patient was found to have both primary adenocarcinoma of the lung and IE DLBCL of the right ventricle. After completing two cycles of IV cisplatin, pemetrexed, and nivolumab, the patient began treatment for lymphoma. She was admitted to the hospital again for therapy induction and completed two cycles of rituximab, etoposide, prednisone, vincristine, cyclophosphamide, and doxorubicin (R-EPOCH). Due to thrombocytopenia, further cycles were temporarily held for four weeks, followed by reinduction with rituximab, cyclophosphamide, doxorubicin, vincristine, and prednisone (R-CHOP). Due to the lymphoma diagnosis, further treatment for adenocarcinoma was held despite inadequate response after the two cycles, and the patient continues to follow up with oncology at the academic institution, along with palliative care for assistance with pain management. As of now, there is minimal treatment response on follow-up imaging, and unfortunate development of bone metastasis, prompting radiation oncology's evaluation for palliative radiation.

## Discussion

This case presents a unique scenario involving the diagnosis of two concurrent primary malignancies: primary lung adenocarcinoma and IE DLBCL of the right ventricle. The concurrence of both cancers is exceedingly rare, presenting diagnostic challenges and a prolonged treatment course.

Primary lung adenocarcinoma is commonly associated with smoking and often diagnosed in an advanced stage due to patients being asymptomatic in the early stages [4]. Advanced imaging techniques, such as CT and PET scans, are vital for detection and staging. In our case, abnormal CT imaging was the first suspicious sign of malignancy. The role of histopathological confirmation through biopsy is crucial for accurate identification and prognostication. Biopsy results of the lung mass were positive for cytokeratin 7 and TTF-1, markers that are highly specific for adenocarcinoma of the lung [8]. Cytokeratin 7 is an intermediate filament protein found in epithelial lung cells, commonly associated with lung adenocarcinoma [8]. TTF-1 is a nuclear protein involved in the development of the lungs and is highly specific for adenocarcinomas. A positive TTF-1 assists with distinguishing adenocarcinomas from other tumor types [8]. Treatment for lung adenocarcinoma often involves a combination of surgery, chemotherapy, radiation, and targeted therapies. Our patient’s regimen of cisplatin, pemetrexed, and nivolumab combined cytotoxic chemotherapy with immunotherapy. Nivolumab, a programmed death-1 (PD-1) inhibitor, has been a particularly significant advancement in NSCLC treatment [9]. The prognosis for lung adenocarcinoma is highly variable, with early detection and targeted therapies improving outcomes. Molecular profiling, such as epidermal growth factor receptor (EGFR) and anaplastic lymphoma kinase (ALK) mutations, is crucial for guiding therapy [10]. Our case's molecular profiling did not reveal EGFR and ALK mutations, which did not offer targeted therapy based on these markers.

In PCL, specifically IE DLBCL, certain immunohistochemical markers are critical for diagnosis. CD45 and CD20 confirm lymphoid origins of the malignancy, while other markers such as MUM1, Pax5, and BCL6 are specific to DLBCL. MUM1 indicates inappropriately active B-cells, while Pax5 suggests early B-cell differentiation [11,12]. BCL6 is a transcriptional repressor involved in the pathogenesis of DLBCL, and overexpression is strongly associated with poorer outcomes [12]. For DLBCL, systemic therapy is often indicated. Our patient initially started on the R-EPOCH regimen, with a transition to R-CHOP due to concerns for tolerance. Studies have suggested that the differences in approach regarding doxorubicin between the two protocols may lead to improved cardiac outcomes. Specifically, R-EPOCH has a prolonged infusion of doxorubicin rather than the bolus found in R-CHOP, which could decrease the risk of toxicity and acute cardiac complications [13,14]. While some studies do suggest improved outcomes in certain populations with the more aggressive R-EPOCH therapy, there are also increased adverse events and issues with tolerance [13,14]. PCL typically has a poor prognosis. The rarity of PCL makes treatment challenging, but there are improved outcomes with early and targeted treatment [15].

## Conclusions

This case highlights the difficulty and complexity of diagnosis and management for patients with more than one primary malignancy. The coexistence of pulmonary adenocarcinoma and IE DLBCL of the right ventricle is exceedingly rare and requires a thorough diagnostic workup and a multidisciplinary team spanning three different hospital systems. Imaging, biopsy, and immunohistochemical markers were vital in accurate diagnosis and targeted treatment for both malignancies. Unfortunately, our patient continues to have treatment-related complications and progression despite medical therapy. Further research and case studies should focus on the complexity of management of similar cases with two or more pathophysiologically distinct malignancies.
